# Observations on the Effects of Opium on the Human Body

**Published:** 1803-12-01

**Authors:** F. Weber

**Affiliations:** Foreign Depot, at Lymington, Hants


### Observations on the Effects of Opium on the Human Body, *by* Mr. F. Weber, *of the Foreign Depot, at Lymington, Hants.*

[ Continued from pp. 435—443. ]

To the first and the latter of the last three men, I administered the following morning at ten o'clock, when they were perfectly well, four grains each, in pills. In half an hour the first perceived a pressure in the forehead. In an hour this pressure was increased to giddiness and slight confusion. At the same time he experienced dryness in the mouth and fauces, and grumbling and contraction of the bowels. At one o'clock he took his usual dinner, being then less giddy than he had been at eleven and twelve. At three he felt no effect whatever.

The other was very giddy and confused an hour after he had taken the pill, he also felt a grumbling and slight pain in the abdomen. His face was at first glowing; he felt a strong pulsation of the arteries in the head. Afterwards his pupils became extremely contracted, and did not dilate either in the dark, nor when I rubbed the eyes with my finger; his sight was dim; his mouth and fauces dry and covered with a tough viscous saliva. Half an hour later he was trembling, sick, and so giddy that he could not walk without a stick. He ate no dinner, and continued in that state, sometimes in bed, sometimes walking about, until the evening. He was in a strong perspiration the whole of the afternoon. At seven in the evening he drank two dishes of tea, which he threw up again, and violently vomited for nearly five minutes. At nine o'clock he complained of violent gripings, against which a quart of warm milk gradually drank, afforded him instant and lasting relief. He continued confused, giddy, very dry, sick, and strongly perspiring the whole night. He did not succeed in making water until one o'clock in the morning, and not before he had made several efforts in vain, and he then perceived a burning in the urethra and frequent interruptions in the stream of urine. He was tolerably well next morning, but had not had a stool since he took the first three grains, three days before.

Livinus Roculé, aged 31, and Küster, aged 20, both enjoying a general good state of health, suffered at the same time violent pains, though the latter in a much higher degree than the first. Roculé from two lymphatic swellings in the knee-joint and groin, in consequence of abruptly drying up some small ulcers of long standing on the leg; the other from a tense erysipelatous swelling of the leg from the same cause.

I ordered each a pill of three grains of solid purified opium, at eleven in the forenoon; which relieved neither the pain, nor did they perceive any other effect. Between seven and eight in the evening, I administered to each five grains in a pill, which perfectly delivered them from pain in an hour's time.

Kûster had a good night, experienced no other effect but dryness in the mouth and fauces, and was perfectly well next morning.

Roculé had also enjoyed rest and sleep; he appeared to be (next morning) in high spirits, and to speak with unusual confidence. He told me, that some time after the pain had left him, he had felt a strong itching in the nose and all over the body, dryness in the mouth and fauces, hoarseness, hickup and thirst; a number of red spots, resembling, according to his description, flea bites, only larger in size and without a tittle in the centre; and though he had not perceived any giddiness or sickness before, he had thrown up his breakfast, which he had taken with a good appetite at seven o'clock. He had also experienced slight difficulties in making water, frequent involuntary stoppings in the stream of urine, and a burning in the fossa navicularis.

Both were regular in their bowels. In Kûster the pain did not return, and the swelling subsided in a few days after the ulcers had again began to suppurate, in spite of art and remedies. To see what effect opium would now have upon him, I gave him three grains (after four days had elapsed since he took the former) three hours and a half after dinner. In about an hour he perceived violent gripings, which lasted till one o'clock in the morning. Two pints of warm milk, which were used three hours and a half after the gripings had appeared, seemed, instead of relieving, to increase them. He experienced not the least giddiness, nor any other effect, and was well next morning.

Roculé, whose ulcers had remained dry, complained again of pain, though not so violent as before, in both swellings, which were highly inflamed; I ordered him three grains, (which was on the fourth day since he had taken the first) four hours after dinner. He found himself relieved in an hour after, though not quite free from pain. He had a tolerable good night, but experienced again much much thirst, and an itching in the nose and all over the body. The pains having rose again next day, I ordered him six grains in a pill, two hours before dinner. In three quarters of an hour he began to perceive some effects in the head, and in another half hour found himself perfectly free from pain. He has continued to take from time to time, four, five, or six grains, perhaps every second, third, or fourth day, which has kept him free from pain, and in good spirits; while, at the same time, the ulcers have remained dry, and the inflammation in the swellings taken its course. The one in the groin has suppurated for some time, through a narrow opening which had formed spontaneously, and seems now going to be dry and to close again. The other in the knee has pointed and is fluctuating. His general state of body appears not to be affected. He is free from fever, his appetite good, and bowels regular.

John Behn, twenty-six years of age, in a general state of health, complained of pain in the wound, four days after I had amputated the large toe of the right foot. I administered to him a pill of three grains in the evening, which perfectly relieved him in an hour's time. In another hour he was giddy, and though very drowsy, had little sleep during the night, on account of frequent startings and disturbed imagination. In the morning he experienced slight difficulties in making water, and threw up his breakfast. At ten o'clock, when I saw him, he was well. Four days after, though free from pain I gave him three grains, three hours and a half after dinner. In about an hour he began to feel a pressure in the forehead, under the tubercula frontalia. In half an hour more the pressure was much increased, he was slightly confused, giddy, and dry in the mouth and fauces. In another hour he complained of pain in the head, above the right arcus supra-orbitalis, which gradually increased, prevented him from sleeping the whole night, and did not quite leave him till the evening of the next day. He was very dry during the first night, experienced frequent startings when he was dosing, and had great difficulty in making water. He had experienced no other affection of the bowels but costiveness.

Cornelius Berlett, thirty years of age, of a healthy habit, was afflicted with a most extraordinary kind of ulcers all over the body, of years standing; the obstinate remains of lues venerea, or possibly of the inconsiderate use of mercury. While some of these ulcers were healing, others broke out again. They were always preceded by hard circumscribed swellings, in and under the skin, which made their appearance suddenly, were inflamed, and extremely painful. He had taken from six to eight grains of solid opium per day, for a great length of time, and perceived but little benefit from it. On my advising to take a larger dose (having some pills constantly near him) he told me, that he could take any quantity without perceiving more effect from it, than from what he usually took. In order to convince me of the truth of what he said, he took in my presence twelve grains. Three hours after, when I saw him again, which was at two o'clock, he had not perceived the least effect. I now administered to him sixteen grains in four pills, and in less than a hour he was free from pain. He continued perfectly easy, and it appeared to me in high spirits, two days, without feeling any other effect but thirst and itching in the nose and over the body the first night. He now takes from eight to twelve grains every second or third day, according to circumstances, is always free from pain and in good spirits, and experiences no effect whatsoever, not even thirst or costiveness. When he first began to take opium he experienced frequent difficulties in making water, and even once or twice a temporary ischuria; and until now he has experienced strong itching in the nose, and over the body, each time when he has exceeded his usual dose. This I state on his authority.

Helfer, aged fifty-six, orderly man in the hospital, had been afflicted with a diarrhœa four days, attended with gripings. He had had about twelve thin stools every day, was free from fever, and had a tolerable good appetite. I administered to him four grains of solid opium, dissolved in a small quantity of brandy diluted with water. Some hours after he found himself free from pain, and had no more stools afterwards. He experienced neither giddiness, confusion, sickness, thirst, nor any other effect whatever. Next morning, at half past eleven, when he was quite well, I gave him, in order to make a comparative observation, three grains dissolved as before. In less than an hour he made a full dinner, and soon after felt giddiness and confusion, a grumbling and contraction in the abdomen, and sickness, which continued till late at night. He was well next morning.

——Weber had laboured fourteen months under a stricture, in consequence of which his general state of health had been considerably impaired. By the introduction of catgut, the size of which I gradually increased, I succeeded in a few days to introduce bougies of a moderate size.

His

His being excessively irritable, obliged me to administer opium in considerable quantities, whilst I was operating on him. Having abruptly discontinued the use of opium, as soon as he could bear the introduction of bougies without pain, I found him, the third day after, in a comatose sleep, which increased the next day to such a degree, that loud calling and shaking only could rouse him. It gradually diminished, and totally disappeared two days after. This happened at a time when I had not made the effects of opium my particular study; and I must confess, that not myself alone, but other medical gentlemen, were alarmed in no small degree, and utterly at a loss how to act. Six months ago he had been in a similar state, and, as was afterwards found, from the same cause.

An unfortunate Frenchman, of the name of Cerisier, twenty-six years old, who has been above a year in the hospital, labouring under a most excruciating anomalous gout, I found in a state resembling a low nervous fever. His countenance was almost hippocratic; his eyes looked muddy, and were filled with a thick yellow matter; he could hardly speak, and the words were indistinctly articulated; he was sunk together in bed like a corpse; could hardly lift up a finger; and was continually dosing. I should have thought this wretched sufferer dying, and in that supposition perhaps have done nothing, (I mean nothing to the purpose) had I not experienced already a like exhaustion on myself, only in a less degree, as the late consequence of abruptly discontinuing the use of opium. I knew he had taken large quantities of opium formerly, (from ten to twenty-four grains per day) and directly guessed, from the change of medical attendance which had taken place some days since, it had been discontinued. Having made enquiries about it, I learned that it was the second day since he had been in this state; that he was worse this than the day before, and that the use of opium had been discontinued five days ago. I ordered him twenty-five drops of tincture of opium, and the same quantity of spiritus ætherius nitrosus, to be taken immediately, and to be repeated once in the afternoon. I did not order him a larger dose, because he was weak and exhausted, and because I would bring those changes gradually about, from which he should derive stronger phenomena of life.

I saw him again several times in the afternoon and evening, but could not perceive any material alteration. At nine o'clock I ordered him forty drops of tincture of opium, and twenty-five of spirit. æth. nitrosus. I gave orders that that he should be left quiet during the night; that he should take a dose (twenty-five drops of each) at six in the morning, and another at nine. At ten o'clock next morning, I thought I could discover a slight increase of strength and energy, and more brightness in the eyes. In the evening, having taken a third dose of forty drops in the afternoon, a visible change had taken place; his eyes had more life; his speech was more distinct; he had more power in his limbs; and less tendency to sleep. I gave orders that the same treatment should be continued. Next day, about noon, I had the satisfaction to find him in the state he had been in before he sunk into this inanition of strength. His eyes were animated; his speech was clear and intelligible; he desired and relished some victuals; and was able to perform the few motions which were not yet impeded by affections of their respective organs.

Another Frenchman, between thirty and forty years of age, apparently strong and healthy, whom a lively imagination disturbed day and night, and in whom the strength of his nerves, unproportionate to the weakness of the other soft solid parts, falsely reported to the sensorium commune, and magnified the least deviation from health in any part of the body, complained every day of a new disorder and affection. In order to blunt the acuteness of his sensations, and if possible raise his spirits, I prescribed to him a grain of opium to be taken four times a day. Not perceiving any effect from this, I doubled the quantity next day, which I soon perceived had the desired effect. His spirits began to raise; sensibility, judgment, and reflection became diminished, and with them his disorders vanished; only, at times, he perceived slight confusion and giddiness; for instance, after dinner and tea, some motion and grumbling in the bowels, thirst and costiveness.

What I have said of myself respecting the circulation of blood, general temperature of body, the processes of the economy at large, and appetite, I have found to be in general the case on those persons on whom I had an opportunity strictly to observe the effects of opium.

If we compare the foregoing observations on persons in health and on persons under disease, we shall perceive a material difference in the effects of opium on them. It will be seen, that, if certain affections were present in the body, against which opium proved to be an effectual remedy, such as fluxes and pain, sometimes uncommon large quantities were requisite to produce the desired effect, if if the said affections prevailed in a high degree, without producing hurtful or hardly any of those effects which much less quantities would have caused in others, or in the same persons, free from those affections. We shall also perceive, that when those affections which had resisted the operations of opium on the body were subdued, the usual effects then would take their regular course, in case a stronger dose had been administered than was required to answer the first purpose.

Mr. B. Weber, Staff Surgeon to the Foreign General Hospital, has frequently been obliged to order from twelve to forty-eight grains of solid purified opium in four and twenty hours, before he succeeded in producing the desired effect: removing pain. He has even been under the necessity, some months ago, to order three grains every hour; so that the patient, who suffered violent rheumatic pains, took seventy-two grains in twenty-four hours, without perceiving hardly any other effect but a removal of pain. It was administered by a trusty apothecary, and partly in our presence. Under this pleasant mode of treatment, the patient was cured in a short time; pleasant, I call it, because the mind being rendered unconscious of what passes within the body, no pain is felt while the disease takes its course.

To men labouring under purulent fluxes, in consequence of affections of the bowels, in the West-Indies, we have also been obliged to administer opium in large quantities, before we could perceive a reduction of stools and removal of pain; and without being able to discover other usual effects of opium, such as confusion and giddiness, difficulties in making water, sickness, &c. Having now stated every observation on this subject which has appeared to me interesting in some respect or other, I shall proceed to take a short review of the whole, extracting all effects, in order to bring them under one point of view

The effects which make their appearance first, are:

Tranquilising passions and emotions, removal of pain, and reducing the frequency of stools. After this is effected, or where the mind was in a tranquil and easy state, and where none of the named affections were present, there appears: sensation of fullness in the head; increased vigour and activity; elevation of spirits; unusual watchfulness; a pressure in the forehead; a slight confusion of thought and giddiness; heat and sensible pulsation of the arteries in the head; flushing in the face; redness and swelling of the eyes; slightly impaired sensibility; dryness of of the mouth and fauces, and thirst; sickness; grumbling and slight contraction of the intestines; hoarseness; headache; increased giddiness and confusion; hickup; higher degree of insensibility; impaired voluntary motion; stammering in speech; propensity to sleep; startings and sudden perspiration all over the body when sleep comes on; stupor; weary eyes and contracted pupils; dimness of sight; trembling; isthuria and dysuria, with frequent involuntary interruptions of the stream of urine; ultlmately, elevation of spirits and watchfulness; costiveness; diminished desire and impaired functions of generation; finally, lassitude, exhaustion, stupor, and sopor.

Under this number, I should presume, the effects of opium, though admitting of a great variety, would be all comprehended.

Having before given the history of my observations on opium, I shall now take a short review of the stated effects; endeavour to trace them to their origin, and offer my opinion on the manner in which opium affects the body.

From an attentive consideration of the effects of opium, extracted from the foregoing observations, it appears to me evident, that opium causes an affection of the intestines and brain, at least an unusual change of the actions of the said organs. Reduction of stools; contraction of the bowels; griping and costiveness, manifest the affection of the intestines; a general disturbance of the intellectual operations, sensibility and voluntary motion in short of those actions and phenomena which result from, or are dependent on the actions of the brain, prove, that an affection of the latter has taken place. Confusion of thought; giddiness; elevation of spirits and unusual watchfulness; happy and frightful visions during sleep, and when sleep comes on; stupor and sopor are unusual, or preternatural intellectual phenomena. There is a diminution of sensibility, that is to say, the mind does not perceive as strongly and speedily as usual the impressions of external agents, and of what is going forward in the body. Passions, emotions, and pain, being different kinds of lively sensations, engrossing the attention of the mind, must become less acute, in proportion as sensiblity becomes diminished, and the intellectual operations impaired. Voluntary motion is impaired; the organs of speech perform their functions with difficulty, in consequence of which, words are indistinctly articulated; the sphincters cannot easily be opened, in order that the contents of their respective spective cavities may be discharged; they refuse the command of the will, because it has lost its power; the stomach, the diaphragm, and other muscles, preter-naturally contract, and thence there ensues vomiting, hickup, startings and tremblings: all clearly shows a disturbed balance between the operations of nervous and muscular power. Pressure in the forehead; heat and sensible pulsations of the arteries in the head; flushings of the face; redness and swellings of the eyes, and head-ache, are symptoms of an accumulation of blood in the head, and leave no doubt of an affection of that organ. Sexual function is impaired or abolished, because the impression on the mind and consequent exertion of the latter, necessary to cause a due excitement in the parts of generation, do not take place.

Lassitude and exhaustion, stupor and sopor, the late consequences of a strong dose, or of suddenly discontinuing the use of opium, prove a languid flow of nervous power; a dullness in the actions of the brain, and the abstraction of a strong exciting cause.

Are not these effects unequivocal symptoms of an affection of the intestines, and, particularly, of the brain? If any doubt remained with regard to the latter, would not the consideration that, by an attentive observation, the first effects are always felt in the forehead, and that only when a large dose has been taken, affections in distant parts take place, but which are all unusual changes of actions and phenomena, in so far as they are depending on nervous influence; would not, I say, this consideration be quite sufficient to convince us that opium chiefly affects the brain, and thence produces the salutary effects of removing pain, and promoting rest and sleep?

I shall proceed to take a short review of the other effects, which being either of less moment, or such as only take place at times and under a particular concurrence of circumstances, it is of less importance, though it may seem more difficult justly to account for them. I shall also consider some circumstances, which have a particular influence on the operations of opium on the human body. Dryness of the mouth and fauces, hoarseness, itching in the nose and over the whole body, I conceive to be symptoms of faulty and inordinate operations of nervous power, in the soft parts of the mouth, fauces, larynx, &c. We shall easily account for the constant attendance of dryness of the mouth and fauces, and hoarseness, on the use of opium, if we consider the number of nerves distributed in the mouth, mouth, &c. and the consequent free communication between the brain and those organs; if we consider, that the same phenomena, (dryness of the mouth, &c.) make their appearance in different passions, for instance, fear and anger, (thence the phrase choaked with anger) and in different cases where the brain is affected. A general perspiration breaking out when sleep comes on, seems owing to an increased want of energy or relaxation of the skin, in consequence of a diminished operation of nervous power. This effect is often perceived when sleep is brought on from any other cause, for instance when we fall asleep after dinner, and thus confirms the manner in which I have accounted for it in this case. Startings, which take place at the same time, are owing to the same cause, and show a disturbed balance between the muscles and their regulating power—nerves. So as the foregoing, does this effect often take place, when sleep comes on from any cause, or if any thing makes a sudden impression on the mind, and gives a check to the quiet ebbing of nervous power, which we express by the term, fright.

Sickness and vomiting taking place, when stupor, great confusion and giddiness, contracted pupils and dimness of sight, difficulties in making water, &c. are present, clearly show that they arise, at least in this instance, from the same cause, in the same manner as the other effects. Vomiting taking place sometimes also when hardly any other effect is to be perceived, seems to be a proof that perhaps from the immediate contact with the opium, in some instances, it may be owing to a change in the living properties of the stomach, or according to the theory of the learned Reil,* in the form and mixtion of its most minute component particles.

Solid or fluid substances exciting vomiting, when taken during the operations of opium, act as powerful stimuli on the disordered state of the stomach, and force it to preternatural contraction.

Continual vomiting after the total evacuation of the stomach, appears to me a sure sign, that it (vomiting) is at least not owing to any remaining part of opium in the stomach.

The effects of opium are the stronger the more early in the day it is taken: during sleep the body is in a different state from that during watchfulness; there is a diminution of action,

* Professor at Halle, in his Archief fur die Physiologie, 1. St. 1. B.

action, an important change of processes takes place, the animal principle, or that something from which excitability, power of action, in short, life results, and which is consumed by every action of the body, accumulates; impressions therefore are more lively after sleep, chiefly on this account, but partly also, the body being less used to them, they are more strongly felt.

Sleep removes slight remaining effects of opium: as the animal principle accumulates during sleep, the different parts must sooner recover their healthy and perfect animalized state again. During sleep also the animo-chemical processes, consequently secretions, are diminished: thus if any part of opium remains in the body, or if considerable changes in the organisation or living properties of the brain, stomach, or other parts, had taken place, requiring strong animo-chemical processes to attain their natural state again, sleep then must prolong the effects of opium.

By the long continued use of opium the effects become less, &c. Various powers which are obnoxious to the health of the body, when applied abruptly, or which would deprive the animal machine of its characteristic properties if they came suddenly, and in a certain quantity, in contact with it, produce, by repeated or gradual application, such changes as not only to become harmless, but even take the place of natural stimuli; that is to say, they become absolutely requisite to excite either single parts only, or the body at large, to actions, without which life could not exist, or be at most but imperfect. Thus it is, that our body becomes inured to various and unhealthy climates; to breath obnoxious gases; to require the use of burning spirits and corroding substances; that the hands of one man become used to work, which would blister another's skin; thus is the organisation of the nose and mouth transformed into new organs of sense by tobacco.

Exercise and heat causing a lively, quick circulation of blood, hasten the operation of opium on the body; but at the same time, causing a particular strong excitement on the surface, thereby lessening the actions of the intestines, and finally hastening the animo-chemical processes, cause the opium to be speedily expelled out of the body. Cold, fear, suspence, and anxiety, lessening the general excitement, and abating the vigour of the processess of the economy, retard the absorption and circulation, consequently the effects of opium on the brain. Cold, if not a very high degree, counteracts the effects of opium less than the said passions, because, though by lowering the excitement on the the surface, and preventing a lively free circulation of blood, it may in some measure prevent a speedy contact of the opium with the brain, its effects are generally of short duration, the same degree soon becoming a relatively stronger stimulus in proportion as the animal principle (Brown's Excitability) becomes accumulated; and because opium affects such organs as are seldom positively affected by cold, and the excitement of which is even increased during the operation of the latter, (in a moderate degree) on account of the blood accumulating in them.

Pain and strong sensations of any kind, seem to cause an exertion of the brain; emotions, such as suspence and fear, an overstraining as it were of the latter, both resisting the operations of opium on that organ. The latter, wholly engrossing the intellectual operations, check or arrest the influx of nervous principle into the body, and thus cause an abatement or a total cessation of most actions depending on nervous influence. A fainting from fright gives us an instance of the latter case; in the first case sensibility is diminished, voluntary motion is difficult, weak and inordinate; breathing is short; and the blood not passing freely through the lungs, and not undergoing there the due changes, excites the heart to irregular contractions; thence faulty secretions and absorptions, cold, clammy, offensive perspirations, indigestion and purging; in short, faulty animo-chemical processes; thus the opium, not being absorbed, lies ineffective in the intestinal canal, and, as it were, out of the system.

As we now see that all effects of opium are symptoms of an affection either of the intestines, or particularly of the brain, and that neither pulse nor general temperature, nor the processes of the economy at large, are directly affected by it, we must conclude that the immediate effects of opium are local, and confine themselves to the said organs, as the effects of jalap to the bowels, ipecacuanha to the stomach, cantharides to the urinal system, and stincus marinus to the parts of generation.

It may be objected, that the usual voluntary motions, such as the functions of the limbs, breathing, &c. are so little impaired, aud do not suffer materially, unless a large quantity of opium has been taken, and unless the effects take place in a high degree. I answer, that those actions only require a single impulse, and take place mostly without intellectual co-operation, and therefore cannot so easily be effected as other actions and phenomena, which require complicated intellectual operations, such as thought, which which are less habitual, more difficultly excited in the natural state, and require more the co-operation of will and premeditation, such as the excretions of urine and fæces, the functions of generation, &c.

The numberless sublime phenomena which are immediately founded on the actions of the brain, and the great variety of actions and phenomena depending on the state of the former, contain the cause why substances, and amongst them opium, affecting the brain, are attended by so many and various affections, and have thence been thought generally to affect the body. Why opium affects the brain particularly, and not the lungs, liver, or any other organ, I believe all we can say (at present at least) is, that it stands in such a relation with the brain as to affect it in such a manner, when it or some of its particles have been absorbed and come in contact with it.

The affection of the intestines appears to me wholly owing to the opium coming immediately in contact with those organs.

With regard to the manner in which opium affects the organs to which it bears a relation, I am inclined to think, from the costiveness, from the glow of the face, the red and swelled eyes, the giddiness and heat in the head, marks of accumulation of blood in the latter, that it simply contracts their minima vascula.

The sensations of pressure and tension in the forehead, attended by an increased flow of spirits, happy reasonings, courage, cheerfulness, &c. the effects of small doses, show an increased action of the brain, and is most likely owing to an increase of tone; in other words, to a slight degree of contraction of the parenchymatous tubes.

Increased energy, exhilaration, watchfulness, *&c.* taking place after the first turbulent effects caused by a large dose have disappeared, prove that the contraction has given way, and is come to that point which was caused by a moderate dose.

Exhaustion and sleep, the last effects, evidently show a dull and languid state of the actions of the brain, in consequence of the abstraction of a powerful exciting cause.

Exhaustion and sleep being the result of a relaxation in the brain, and this relaxation the consequence of the foregoing contraction, will easily explain why they are proportionate, in degree and duration, to the former effects; why they make their appearance the sooner the smaller the dose had been, the later the larger it was, and the longer the use of opium had been continued. If, for instance, stance, two grains of solid opium had been taken at eight in the morning, a higher or less degree of confusion, giddiness, propensity to sleep, &c. had lasted from three to six hours, and proportionate degree of energy, exhilaration, &c. succeeded, a slight exhaustion may make its appearance in the evening. If in consequence of a larger dose, more violent effects had been produced, and lasted a whole day or longer; if the succeeding exhilaration had been proportionate in duration, it will be obvious that the exhaustion, which cannot make its appearance before the foregoing effects have vanished, will not take place until thirty-six or forty-eight hours have elapsed.

That opium affects the brain in no other way but by being absorbed, and coming in contact with it, seems to prove the late appearance of the effects, which do not take place before at least half an hour has elapsed, the appearance of the effects being hastened by every thing which accelerates the circulation of blood, and increases the vigour of the animo-chemical processes; such as exercise, heat, food, spirits, &c. On the contrary, the appearance, of the effects being retarded, under circumstancs which lessen the actions of the body, and abate the processes of economy, such as cold, rest, sleep, fear, &c. The effects taking a quick course, and speedily vanishing under an increased operation of the processes of the economy, thus expelling the opium quickly out of the body; as, for instance, during exercise, when the body is warm and perspiring. On the contrary, the long duration of the effects, when the processes are slow, as is the case when the body is under the influence of cold, of those passions known by the name of depressing as fear, &c. During rest and sleep, &c. the speedy appearance and higher degree of the effects after a meal, increasing the absorption of the opium in the primæ viæ, and pushing, as it were, that which is in the lacteals forward into the circulation; perhaps, also, the chyle, floating in the blood, and not yet perfectly homogenated, increases the affection of the brain, when it comes in contact with it.—I shall conclude this with a few practical hints, deduced from the whole.

1. Knowing that opium produces the salutary effects, removal of pain, rest and sleep, by causing certain changes in the brain, and thereby rendering it unable to perceive what is going forward in the body, and that the body at large is not *directly* affected by it, nor any other part except the intestines, we shall, *without fear of imparting any morbid*

*morbid state to any part,* the said organs excepted, order whatever dose be necessary to produce the desired effect.

2. Knowing that the effects of opium are the stronger, the weaker the body is, and the more the functions are disordered, provided the parts on which it operates possess a sufficient degree of excitability to receive its impression, we shall regulate the dose accordingly.

3. Lively sensations and emotions resisting the operations of opium on the body, in proportion to their degree, will assist us in determining the proper dose. We have seen above, what uncommon large quantities were required to produce ease in persons labouring under violent pain, and what different effects the same dose produced in the same persons, when labouring under pain, and when free from it.

4. If it be our object to excite lively actions in the brain, in order to produce an increased flow of nervous exciting cause, and thence an increased energy, activity, &c. we shall order from one to three grains or more for the first time, and continue with half a grain, a grain, or a grain and a half, or more, to be taken from every half to every two hours, according as the effects make their appearance.

5. If it be necessary to hasten the operations of opium on the body, we shall administer it dissolved in any usual menstruum, or order at the same time a small quantity of spirits, or any fluid containing alcohol; and in about half an hour after, some fluid or solid substance, as will be judged most adapted to the case.

6. Should a quantity have been taken from which dangerous effects are to be feared, speedy evacuation of the stomach, by mechanically irritating the orifice of the pharynx, will prove the surest means of safety; lemon juice, or where that cannot be procured, vinegar, or I should suppose any other vegetable acid, or sour fruit, taken in as great a quantity as the stomach will bear, will in a great measure render ineffectual that which lies in the intestinal canal. Heat, exercise, or any thing that tends to increase the animo-chemical processes, and causes sweat, will be the most effectual means to free the body from what has been carried into the circulation.

7. To rouse a patient from sopor, occasioned by abruptly discontinuing the use of opium, it will be easily conceived, that the latter must be renewed. The quantity which is necessary to produce this effect will be best determined by the effects produced from a given dose. The degree of sopor, the former state of the patient with regard to strength and weakness, and the quantities formerly taken, may assist in determining the proper dose.

8. In an alarming degree of sopor, being the direct consequence of a large quantity of opium, attended by coldness, insensibility, &c. sprinkling the regio hypogastrica and parts of generation with water, as cold as possible, or a mixture of vinegar, nitre, and ammoniac, will, as one of the most powerful excitantia, deserve particular notice.

The following history of two analogous cases may not be unacceptable, though not exactly belonging to the subject.

At a time when I officiated in the Shropshire militia regiment in the Isle of Wight, for the surgeon who was ill, I was called to two men, privates of the said regiment, who were lying on the ground in a state of asphyxia, in consequence of the immoderate use of brandy. They appeared totally bereft of sensibility, were cold, and without pulse; breathing and pulsation of the heart were only perceivable in one, and not without the nicest attention. I had them undressed, wrapped in warm blankets, placed near a large fire in a horizontal position, yet so that their heads were elevated farthest from the fire, and under a free access of air. I had them rubbed with warm wood ashes, and brushed with hard brushes. After this process had been continued for about a quarter of an hour, I cautiously washed their heads with cold water and vinegar, and sprinkled the before mentioned mixture from a certain height on the said parts, which instantly produced deep inspiration, sighing, and other strong symptoms of life.

This was at twelve o'clock at night, after they had been lying from four or five o'clock in the morning on the beech, where they had found a cask of brandy, and emptied it, in company with eight or ten others.

About two scruples of white vitriol poured into each, produced no effect till they were warm, and showed strong symptoms of life.

